# Clinical Behavior of the Gingival Margin following Conservative “Coronally Dynamic” Restorations in the Presence of Non-Carious Cervical Lesions Associated with Gingival Recession: A Pilot Study

**DOI:** 10.3390/dj10070132

**Published:** 2022-07-13

**Authors:** Felice Femiano, Rossella Sorice, Rossella Femiano, Luigi Femiano, Ludovica Nucci, Vincenzo Grassia, Marco Annunziata, Andrea Baldi, Nicola Scotti, Livia Nastri

**Affiliations:** 1 Multidisciplinary Department of Medical-Surgical and Dental Specialties, University of Campania, Luigi Vanvitelli, Via De Crecchio 6, 83138 Napoli, Italy; rossellasor@libero.it (R.S.); rossella.femiano@libero.it (R.F.); ludortho@gmail.com (L.N.); vincenzo.grassia@unicampania.it (V.G.); marco.annunziata@unicampania.it (M.A.); livia.nastri@unicampania.it (L.N.); 2 Department of Surgical Sciences, University of Turin, 10124 Torino, Italy; andrea.balbi.od@gmail.com (A.B.); nicola.scotti@unito.it (N.S.)

**Keywords:** non-carious cervical lesion, creeping attachment, gingival recession, BOPT

## Abstract

Gingival recessions (GR) are often associated with the presence of non-carious cervical lesions (NCCL). The latter result in the disappearance of the cement–enamel junction (CEJ), with consequent difficulties both in measuring the recession itself and in performing root coverage techniques. The restoration of cervical lesions is consequently an important aspect in the treatment of GR, with the re-establishment of a “new” CEJ. This pilot study aimed to verify whether restorative therapy alone, with the execution of a restoration that mimics the convexity of the natural CEJ and thanks to a slight horizontal over-contour, can stabilize a clot in the intrasulcular site and consequently is able to change the position of the gingival margin in a coronal direction. In periodontally healthy patients, with a non-thin gingival phenotype, 10 GR-associated NCCL restorations were performed using a protocol inspired by concepts of prosthetic conditioning, with a progressively reduced convexity (“coronally dynamic restoration”) and de-epithelialization of the gingival sulcus. We observed that 70% of the treated teeth showed a reduction in crown length after 15 days (−0.267 mm), without an increase in probing depth. While considering the limitations of the sample and the need to evaluate the different parameters that can affect the result, the coronally dynamic restoration of NCCL with GR was able to influence the position of the gingival margin in a coronal direction.

## 1. Introduction

Non-carious cervical lesions (NCCLs) are lesions located at the cement–enamel junction (CEJ) characterized by the loss of dental hard tissue, which can involve enamel, dentin and/or cement, in the absence of an infectious component attributable to carious pathology [1,2,3].

Recent studies suggest that the onset and progression of NCCLs present multifactorial components [3,4,5]. Three main mechanisms are hypothesized as responsible for the onset of NCCLs: bio-corrosion, abfraction, friction and/or abrasion. The prevalence of one of the three processes conditions the morphology of cervical lesions [3,6].

In the abrasion process, it is known that “incorrect” oral hygiene practices are also of particular importance [7], with a preferred localization on the buccal aspect, especially on the first premolars, followed by canines, incisors and molars [8,9].

NCCLs are often associated with pathological conditions related to the loss of structural integrity in the cervical region of the tooth [7], such as:

- dentinal hypersensitivity [10,11];
- accumulation of biofilm, with risk of caries and pulpitis;
- aesthetic deficits;
- gingival recessions [12,13].

The finding that the sites of greatest predilection for GRs are the same as those of NCCLs explains why in clinical practice it is not unusual to find the presence of GRs combined with NCCLs. On the other hand, the exposure of the root surface to the oral environment in the presence of gingival recession, increases the possibility that part of the dental hard tissue undergoes destruction with an outcome of NCCL [14].

Tooth surface defects associated with gingival recession have been classified by Pini Prato et al., highlighting the importance of the CEJ, both for the assessment of the extent of the recession and for the positioning of the surgical flap in corrective procedures. The same authors also underlined the importance of wear/abrasion/erosion (with the creation of a step) that can occur in that same cervical area [15,16].

Zucchelli G. et al., in 2011 classified NCCLs combined with GRs in five different types and suggested the most effective approaches to their resolution, with conservative, conservative and surgical or only surgical approaches [12].

Corono-radicular NCCLs, with the disappearance of the anatomic CEJ, are the most complex lesions, and their ideal treatment consists in a combined restorative–periodontal approach [17,18] in which the restoration of the CEJ should precede mucogingival surgery.

Recently, Derchi G. and Loi I. proposed a new approach for the treatment of NCCLs combined with Miller type I and II GRs, defined by the authors as an alternative to mucogingival surgery with coronal flap displacement [19]. This is a minimally invasive conservative technique that aims to apply the principles of the biologically oriented preparation technique (BOPT) to modify the gingival parables [20].

On this basis, our pilot study was aimed to assess the clinical behavior of the marginal gingival tissue in NCCLs associated with GRs, when treated by a modified restorative method (defined hereafter as “coronally dynamic” restoration), consisting in a slightly “over-contoured” emergence profile of the cervical restoration at the CEJ level and a delicate “curettage” of the marginal gingiva on its internal sulcular side (“gingitage”).

The null hypothesis was that the conservative coronally dynamic restoration technique, according to the principles of the BOPT, had no influence on the level of the coronal margin of the gingiva.

## 2. Materials and Methods

### 2.1. Sample Selection

For our study, patients referring to the Conservative Dentistry Unit (Dental Materials Section) of the Dental Clinic of the University of Campania “L. Vanvitelli”, between the ages of 18 and 65 years, with no gender predilection, were selected.

All patients diagnosed with NCCLs underwent a detailed anamnestic investigation in order to be able to detect and eliminate or correct possible causal factors highlighted in their eating habits (constant use of acidic foods or drinks such as soft drinks, Coca Cola, fruit juices, citrus fruit juices, etc.), in the habitual use of acid-forming drugs (tranquilizers, antipsychogenic drugs, etc.), in their clinical history or after specialist consultation for esophageal reflux diseases (GERD), in the presence of alimentary or nervous vomiting or incorrect oral hygiene practices.

After the screening examination, and regardless of patients’ willingness to participate in the study, the patients underwent a professional oral hygiene session with ultrasonic instruments and polishing of the dental surfaces with a rubber cup and low-abrasion prophylaxis paste. The patients were then instructed to use a soft brush roll technique in sites with NCCLs and/or gingival recessions. The restorative treatment was not performed until the patients demonstrated adequate oral hygiene and the disappearance of traumatizing habits.

The lesions included were NCCLs in association with GRs, present on the buccal surface of permanent teeth of the anterolateral sector (incisors, canines and premolars).

We excluded dental elements that, in addition to NCCLs, simultaneously exhibited:

- carious lesions on the buccal surface or interdental restorations
- or absence of pulp vitality
- or prosthetic crown
- or fractures of the dental crown
- or malposition of the tooth
- thin gingival phenotype
- periodontal pockets and/or tooth mobility.

The gingival phenotype was evaluated by probing the sulcus: when the periodontal probe was visible through the gingival tissue, the gingival phenotype was considered thin, when not visible, it was considered thick [21]. Dental elements with a thin gingival phenotype were excluded from the study.

All recruited patients signed a consent after being informed verbally and in writing of the purpose of our study.

For this pilot study protocol, a sample size of 10 teeth was established.

All the procedures of this study were carried out in accordance with the national and institutional standard ethics validated on animal and human experimentation, which follows the declaration of Helsinki of 1975 revisited in 2000 and therefore approved by the local Ethics Committee of the AORN of the ‘Colli Hospital’ with N° 663 of 09/16/19.

### 2.2. Operative Protocol

**Figure 1:**
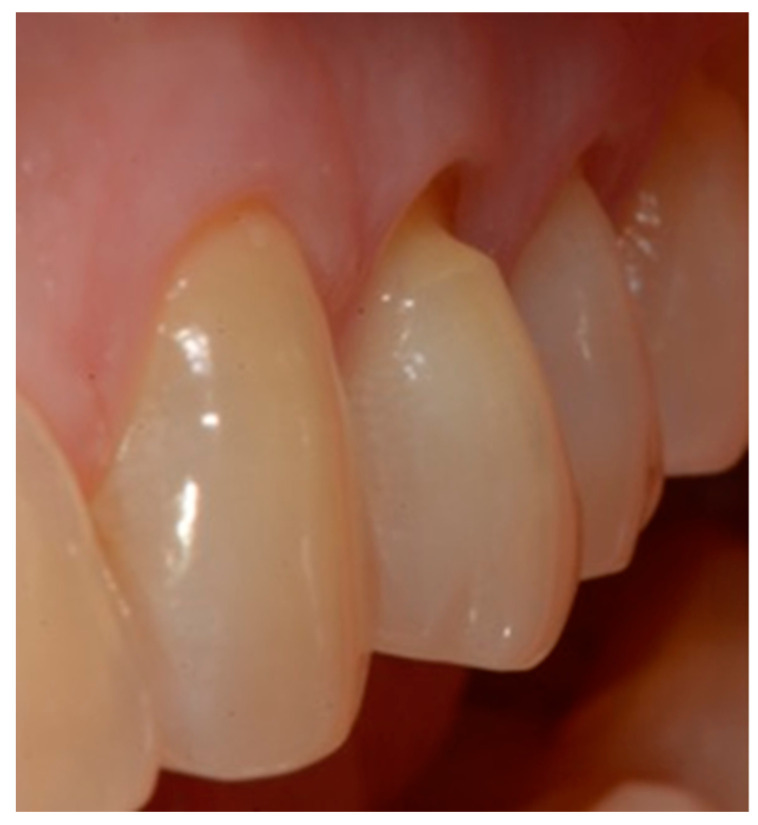
Tooth before restoration.

**Figure 2:**
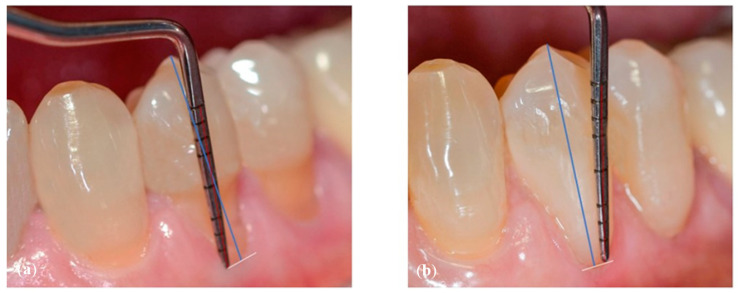
Crown Length (CL) measurement (**a**) at baseline (t0) and (**b**) at reevaluation (t1).

- Selection of the shades (dentinal masses and enamel) of the tooth to be restored (Figure 1);
- Initial measurement of the length of the clinical dental crown (performed with a millimeter periodontal probe placed adjacent to the dental crown and having as a reference the most apical part of the gingival sulcus and the highest part of the crown) and photography of the lesions at time 0 (t0) (Figure 2a).
- Evaluation of the vestibular probing depth (PD) at baseline, by a millimetered Williams probe inserted in the sulcus on the buccal aspect.

#### 2.2.1. Stages of the Restoration

**Figure 3:**
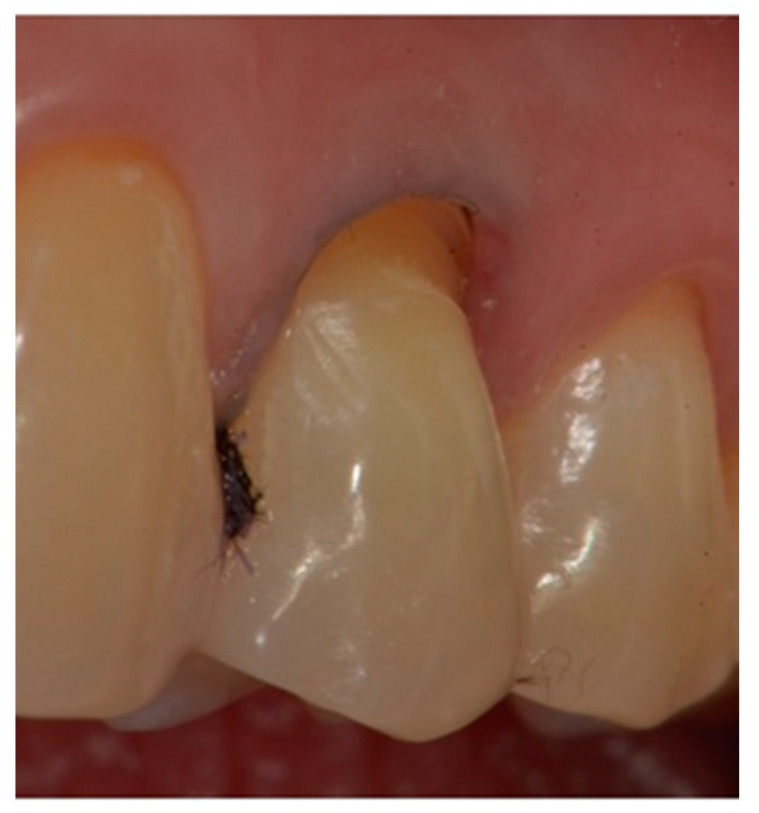
A retraction cord inserted in the gingival sulcus to expose the apical margin.

**Figure 4:**
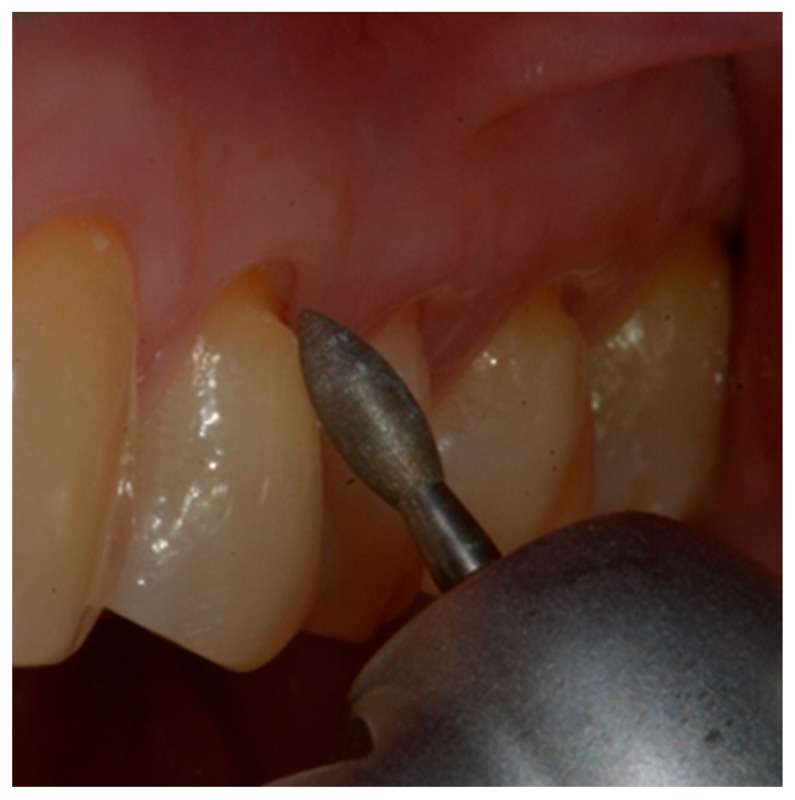
A small bevel was performed on the coronal margin of the NCCL.

**Figure 5:**
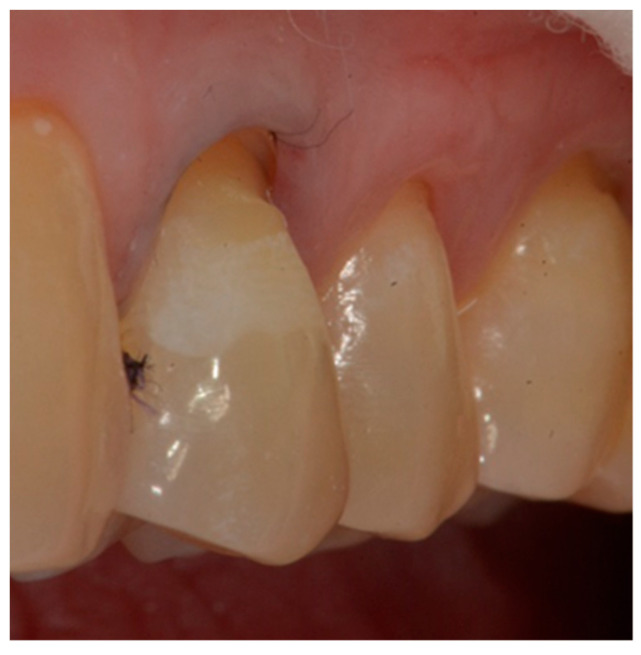
NCCL after etching.

**Figure 6:**
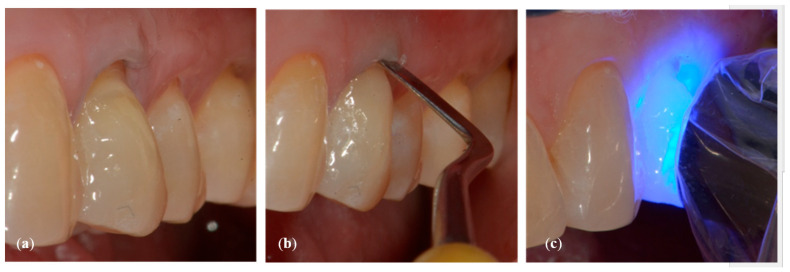
(**a**) A thin layer of flowable composite was applied; (**b**) a paste composite was applied with a convex profile; (**c**) every layer of the composite was polymerized for 20 s.

**Figure 7:**
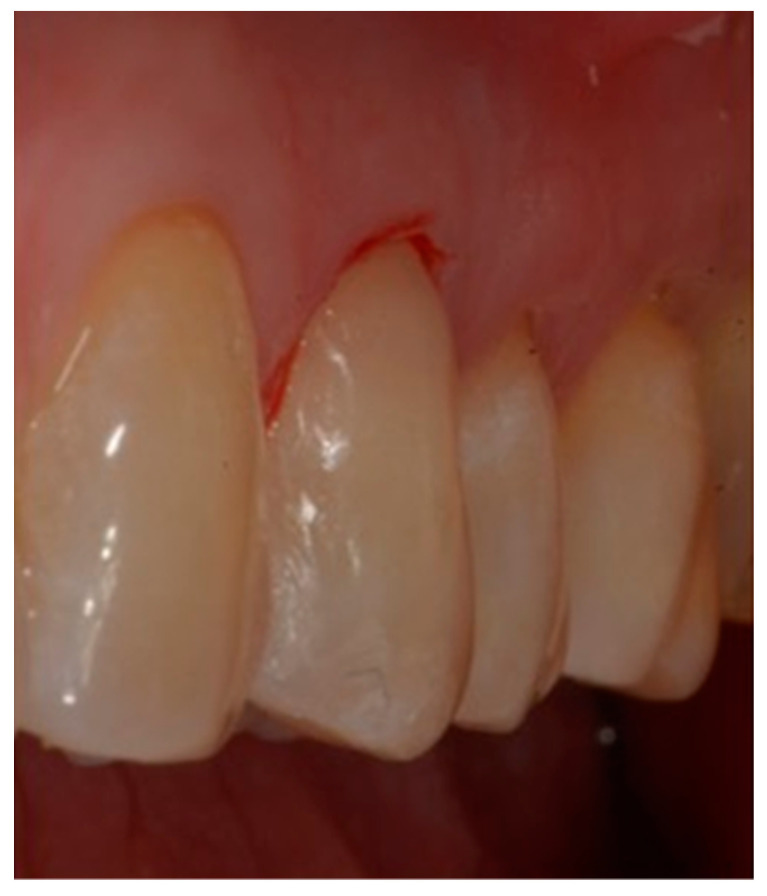
First refinement of the restoration and removal of the retraction cord.

**Figure 8:**
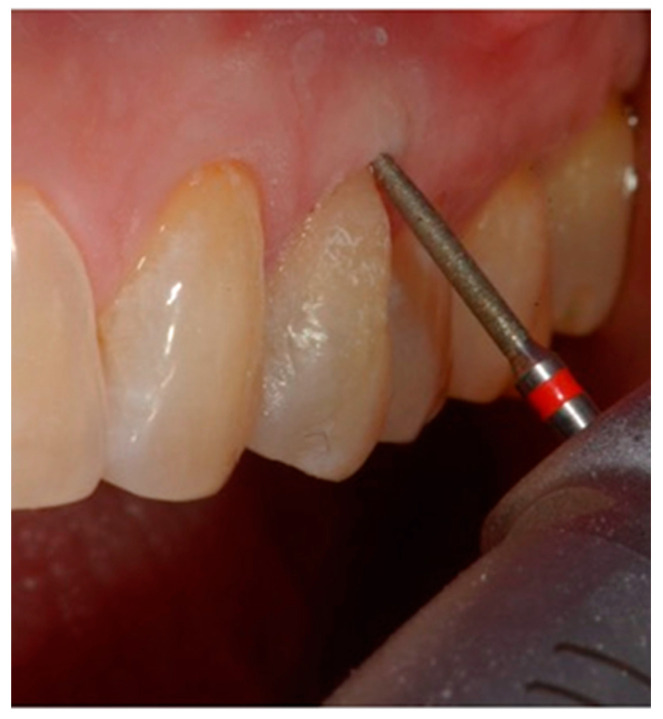
Finishing of the restoration with a flame bur with a 45° angle.

- Isolation of the field with a retraction cord 000 in the gingival sulcus (Ultrapak; Ultradent, 505 West Ultradent Drive South Jordan, UT, USA) (Figure 3).
- mechanical cleaning with a diamond bur of the bottom of the cavity to expose new dentin.
- definition of a small bevel of the enamel (mini-chamfer) in correspondence with the coronal perimeter of the lesion with fine-grained diamond burs to favor the adhesion processes and the mimicry of the restoration and to give sufficient thickness to the most coronal composite (Figure 4).
- 3-step enamel-dentin bonding technique: use of 37% orthophosphoric acid (Axia Etch PRECISION) and primer and bonding (Optibond FL, KERR ITALIA SRL,Via Passanti 332 84018 Scafati, Italia) in multilayer application followed by 20 s of light-curing with an LED lamp [22] (Figure 5).
- Application of a thin layer of flowable composite (3M Filtek Supreme flowable, 3M Italia Srl,Via Norberto Bobbio, 21, 20096 Pioltello (MI) Italy) on the bottom of the cavity and of composite (3M Filtek Supreme XTE, 3M Italia Srl,Via Norberto Bobbio, 21, 20096 Pioltello (MI) Italy), on top and perimeter seal, by means of a layering procedure. The composite was applied in horizontal excess following the principles of the BOPT technique, trying to emphasize the cervical convexity. The polymerization phases were performed using the KERR (KERR ITALIA SRL,Via Passanti 332 84018 Scafati, Italia) 800 Watt lamp for 20″ for each layer [23] (Figure 6a–c).
- First refinement of the restoration and removal of the retraction cord (Figure 7).
- Finishing the restoration with a coarse- (80 μm) and a fine- (50 μm) grain flame bur placed on the most apical point of the restoration, without touching the dental tissue with the tip, in order to create an angle of approximately 45° and maintain a convex profile (Figure 8).

**Figure 9:**
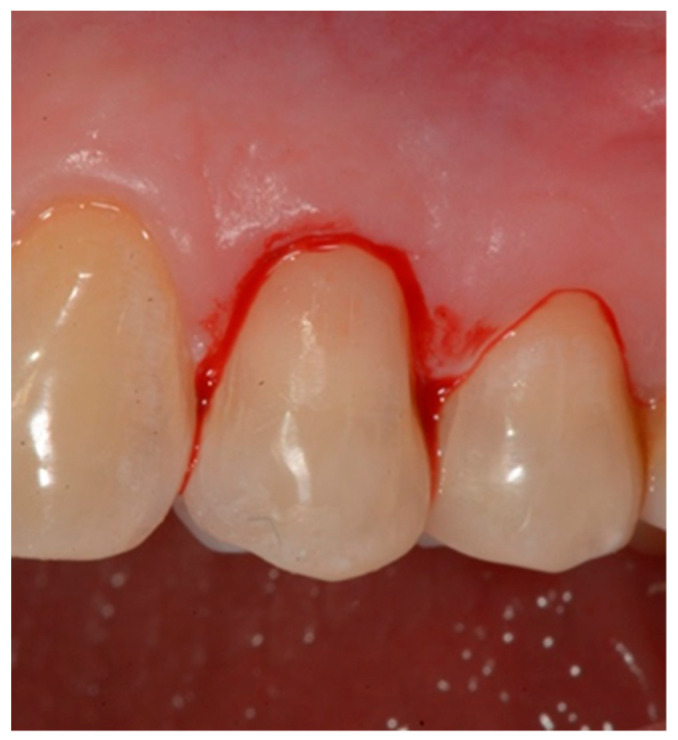
De-epithelization of the sulcus until bleeding was performed.

This procedure was used to maintain a profile with a slight horizontal over-contour and to produce a delicate “gingitage” in the internal side of the sulcus (Figure 9), i.e., to de-epithelialize the sulcular side of the marginal gingiva, without interfering with the connective tissue. The purpose of the gingitage was to determine the formation of a stable blood clot, protected by the restoration convexity.

- Polishing the restoration with fine-grained 50 μm diamond burs, composite polishers and a brush with bristles impregnated with silicon carbide to give the restoration a perfect shine.

All the restorative procedures were performed by a single clinician.

At 2 weeks (time t1), a check of the restoration was performed, and the health status of the surrounding marginal gingiva was assessed by periodontal probing with a Williams probe. PD was measured in the gingival sulcus at the vestibular aspect of the tooth.

**Figure 10:**
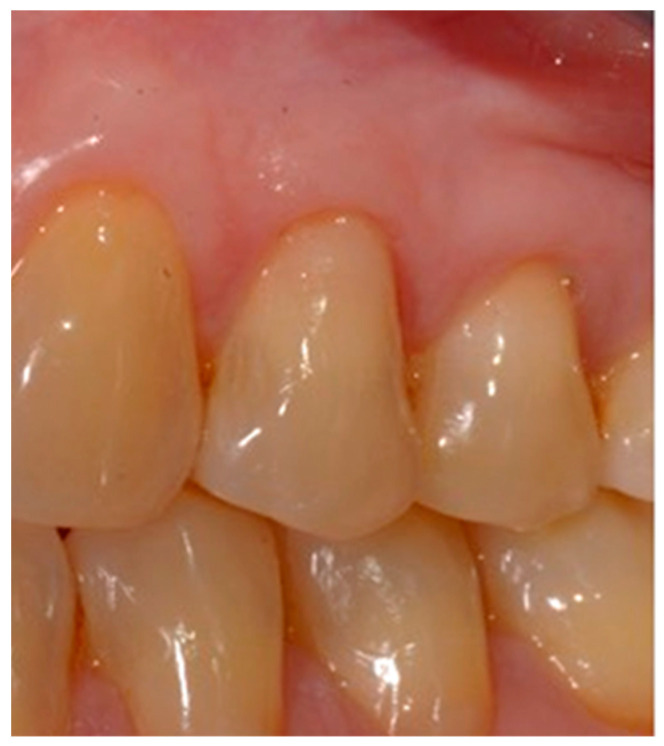
After 15 days from the restoration, the profile was reduced to allow a more coronal gingival adaptation.

At physical examination, the gingival conformation obtained was assessed, and, if necessary, further removal of a small portion of composite from the apical margin of the restoration was carried out, proposing the same 45° inclination and inducing a new gingitage. The purpose of this procedure was to allow a progressive displacement of the convexity of the restoration (referred to as a “coronally dynamic” restoration), the margin of which ideally represented the new CEJ, in a more coronal sense, favoring the progressive adaptation of the gingival margin (Figure 10).

#### 2.2.2. Measurement of Clinical Crowns Length

For each dental element selected for the co-presence of NCCL and GR, the measurement of the length (mm) of the clinical crown (Crown length, CL) was carried out before the restorative treatment (time t0) and 15 days after the end of the treatment (time t1). The difference between these CL values was used as a benchmark to evaluate the migration of the marginal gingiva following the restorative treatment (Figure 2a,b). The modification of the position of the CEJ obtained from the restoration did not allow us to use the gingival recession as a parameter for the evaluation of the position of the gingival margin.

**Figure 11:**
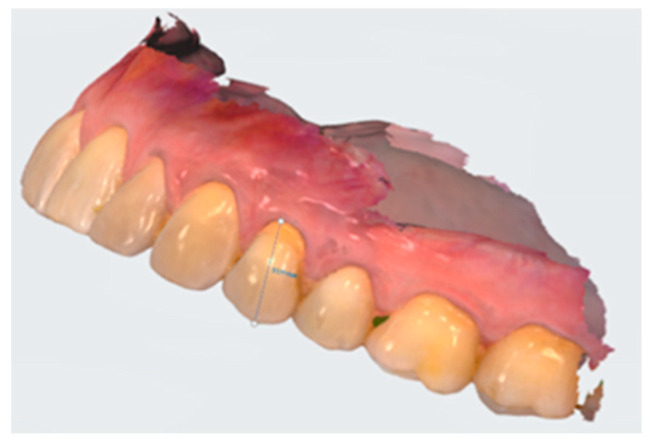
Digital measuring of the crown length on the digital intraoral scan.

The CL estimate was performed on photographic images with standardized framing that portrayed the dental element being analyzed and the probe. For some of the procedures, an intraoral scan with a 3shape scanner (TRIOS3) and differential measurements analyzed with Trios Patient monitoring (3Shape, A/S Holmens Kanal 7 1060 Copenhagen Denmark) software were performed, in order to validate the photographic and analysis procedures with software (Figure 11).

The CL value was obtained by considering the distance between the most coronal end of the clinical crown of the selected dental element (landmark a) and the tangent to the most apical end of the marginal gingiva (zenith) (landmark b). To increase the accuracy of the measurement of the distance between landmark a and landmark b, five measurements were made for each dental element examined (M1–M5), and their mean and standard error were then estimated. For each dental element, the difference (δz) between the average value of the CL evaluated at time t0 (μ0) and the average value estimated at time t1 (μ1) was calculated.

The measurements were performed using the Adobe Illustrator software (10.1.24. version), which allows o performing measurements on photographic images with a precision to the fourth decimal place.

A paired-sample t test was then performed to assess whether the mean value of CL and probing depth at time t0 differed significantly from those estimated at time t1.

## 3. Results

The study sample for our pilot analysis consisted of 10 combined lesions (NCCLs associated with GR), in 7 patients with a mean age of 46 years (±4.69). The average values (μ) and standard error (ε), estimated on repeated measurements of the CL of the dental elements selected for each patient and measured at time t0 and time t1, are reported in Table 1.

In 70% of the study sample, the average length of the clinical crown of the selected dental element (μ1) was reduced after 15 days from the restorative treatment (time t1) compared to the average value (μ0) estimated in the pre-treatment phase (time t0). This percentage index was obtained by adding the events in which the value of δz was positive to the total number of events reported in the last column of Table 1.

**Figure 12:**
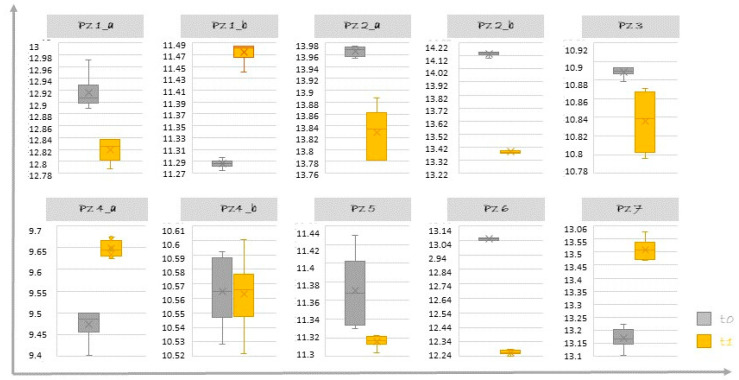
Comparison at t0 (baseline) and t1 (15 days post final restoration) of Crown Lengths (CL) distribution. On the ordinate axis, CL is expressed in millimeters.

This decrease in the CL value is shown in Figure 12 in which the distributions of the CL values carried out at time t0 and at time t1 for each individual dental element are compared by means of box plots (Figure 12).

The mean CL reduction value at 15 days was 0.267 mm (min–max: 0.002–0.782 mm) and was estimated considering the distribution of positive δz values.

Although the difference in CL was evident for some dental elements (Figure 12), it was not statistically significant (*p* = 0.88).

The buccal probing depth of the dental elements subjected to the restorative procedures did not substantially vary (t0: 0.95 ± 0.36 mm and t1: 1.1 ± 0.39 mm), showing a non-statistically significant difference (*p* = 0.343).

## 4. Discussion

In clinical dental practice, the finding of NCCLs combined with GR is increasingly frequent, especially in patients over the age of 41 [13]. When these lesions are restored, the clinician has to pay attention to the etiology, which should be thoroughly investigated, before replacing what has been lost [6], i.e., the morphology of the dental and gingival tissue. The efficiency of the treatment is in fact strictly correlated with the identification of the multiple etiological factors of these lesions.

For the treatment of these combined lesions, the choice of the clinician can be oriented towards periodontal plastic surgery, a traditional restorative approach, specifically aimed at the treatment of hard tissues, or combined surgical–restorative treatments. These techniques have been widely validated and documented by literature data [12].

The rationale of the proposed coronally dynamic restoration can be found in the literature in the so-called “restoration-guided creeping attachment” [19,24], and is based on the hypothesis that the restorative treatment of an NCCL, if carried out by recreating the characteristic cervical convexity of the natural dental element as established by the BOPT technique, can modify the gingival tissues attached to it (concept defined as prosthetic dominance in the discipline of dental prosthesis). Therefore, also in the restorative treatment, the expected epiphenomenon would be represented by the modification of the gingival tissues, recognizable in an increase in the thickness of the gingival tissue on the horizontal plane and in its migration in a more coronal position on the vertical plane [25,26,27]. Our pilot study tried to validate the clinical possibility that this coronal migration occurs following restorative procedures that involve the creation of an intrasulcular space for the clot, made stable by the restoration itself.

For the new position to be acquired by the gingival tissue, the role played by the new CEJ, recreated with the restoration, is fundamental. This new CEJ is already widely used in combined restorative–mucogingival procedures to ensure a point of anatomical convexity on which the coronally displaced flap can find stability and seal against the clot or an underlying connective graft.

The aim of our study was to verify whether, in the absence of a flap, the restoration alone was able to determine a change in the position of the free gingival margin. The results seem to confirm that a correct morphology of the restoration, obtained through the creation of a new CEJ, with a slight final convexity and accompanied by slight de-epithelialization of the sulcus, in the healing phases may be able to determine a creeping attachment in the coronal direction, of a minimal entity, often variable between subjects and within subjects, in relation to factors not yet known. However, the results of the study open to a possible application to NCCL and GR, especially in subjects who do not accept or cannot undergo periodontal plastic surgery.

Our results can only be compared with some data present in the literature, mainly case reports or case series; however, the presented protocol differs in some phases from those used in previously studies [20,27]. In the work of Derchi G. et al., [19], the authors introduced a preliminary phase to the restoration, which was absent in our procedure. After smoothing the exposed root surface, the authors slightly surgically detached the marginal gingiva at the recession level and inserted a collagen sponge apical to it, before starting the restorative procedure. In the case report by Perelli M. et al., the possibility of observing the creeping attachment on natural teeth following a non-surgical approach based only on the de-epithelialization of the sulcus and the upper part of the junctional epithelium with a diamond bur (grain size 120 μm) was described [24]. In fact, after a follow-up of 24 months, the authors reported a coronal margin growth greater than 2 mm. These data support the hypothesis that the coronal migration that we clinically observed in our analysis sample can be explained by the phenomenon of creeping attachment.

In the prosthetic field, in the study by Rodríguez X et al. [28], the authors presented the first study in which a histological examination was performed in humans (periodontal ligament of two dental elements) to evaluate the response of the tissues to vertical preparation and immediate provisionalization with the BOPT technique. The reported results showed that it would be possible to obtain a regeneration of the connective tissue along the surface of the dentin of the previously prepared tooth, which would explain the clinical stability of the periodontal tissues obtained with this technique. However, histological studies in humans carried out on periodontal tissue adjacent to conservative treatments of cervical lesions associated with gingival recessions are lacking.

The literature reports the possibility of treating recessions <2 mm with a non-surgical approach [29], which includes the polishing of the exposed root tissue with curettes, together with the modification of the patient′s oral hygiene maneuvers. However, the creeping attachment that can be obtained in these cases is severely limited in the presence of NCCL. The concomitance of NCCL and GR often requires a combined approach (conservative and surgical) that not all patients are willing to accept. With this in mind, the possibility of obtaining a coronal migration of the gingival margin following a minimally invasive conservative restoration procedure could represent a therapeutic alternative worthy of further study.

In fact, the present study was intended as a pilot study, aimed at verifying the possibility of obtaining a variation of the cervical margin following a simplified conservative restoration procedure associated with “gingitage”. There are some aspects that need to be highlighted and that will require further verification. From the observation of our data, it is appears that there was a wide intra- and inter-individual variability. The study presents some limitations: due to the small sample size, it was not possible to assess the predictability of the technique and all the parameters that affected the expected results; in addition, the short follow-up did not allow a long-term verification of the stability of the results. It has also to be considered that our study selected only NCCLs with recessions and a “non-thin” gingival biotype. Although the technique has also the scope of thickening the gingiva, performing a de-epithelization in a thin gingival biotype (where the sulcus has a very thin layer of connective tissue between the inner and the external layer of the epithelium) could pose a severe risk of development of gingival recessions. At last, the proposed technique could be technically demanding and operator-sensitive.

## 5. Conclusions

The literature supports the hypothesis that a biological basis exists that makes possible the potential modification of the gingival architecture following the principles of the BOPT also in conservative restorative dentistry, but there are still no consolidated data on the predictability of this technique. The sample we used in this study represents the largest so far described in the literature on which this technique has been tested. Within the limits of the study, the application of a restoration with adequate cervical convexity and determination of a stable intrasulcular clot was shown to induce coronal migration of the gingival margin in the presence of a non-thin gingival biotype, non-carious cervical lesions and gingival recessions.

## Figures and Tables

**Table 1:** Descriptive statistics of crown lengths (CL).

NCCL Number	Baseline Measurements (t0)					Reevaluation Measurements (t1)					Mean, μ		Standard Error		Mean CL Variation
	**M1**	**M2**	**M3**	**M4**	**M5**	**M1**	**M2**	**M3**	**M4**	**M5**	**t0** (**μ0**)	**t1** (**μ1**)	**t0** (**ε0**)	**t1** (**ε1**)	**δz**
**1**	13.0	12.9	12.9	12.9	12.9	12.8	12.8	12.8	12.8	12.8	12.9	12.8	0.014	0.010	0.095
**2**	11.3	11.3	11.3	11.3	11.3	11.5	11.5	11.4	11.5	11.5	11.3	11.5	0.004	0.008	−0.188
**3**	14.0	14.0	14.0	14.0	14.0	13.9	13.8	13.8	13.9	13.8	14.0	13.8	0.004	0.021	0.137
**4**	14.1	14.1	14.1	14.1	14.1	13.4	13.4	13.4	13.4	13.4	14.1	13.4	0.008	0.005	0.751
**5**	10.9	10.9	10.9	10.9	10.9	10.9	10.8	10.9	10.9	10.8	10.9	10.8	0.003	0.015	0.053
**6**	9.5	9.5	9.5	9.4	9.5	9.6	9.6	9.7	9.7	9.6	9.5	9.6	0.019	0.011	−0.174
**7**	11.4	11.4	11.4	11.3	11.3	11.3	11.3	11.3	11.3	11.3	11.4	11.3	0.019	0.003	0.054
**8**	10.6	10.6	10.6	10.6	10.5	10.6	10.6	10.5	10.5	10.6	10.6	10.6	0.012	0.013	0.002
**9**	13.2	13.2	13.2	13.1	13.2	13.6	13.5	13.5	13.5	13.5	13.2	13.5	0.020	0.020	−0.339
**10**	13.1	13.1	13.1	13.0	13.0	12.3	12.3	12.3	12.2	12.3	13.0	12.3	0.004	0.008	0.782

Descriptive statistics of crown lengths (CL) of the analyzed teeth (five measurements per tooth, M1–M5) at t0 (baseline) at t1 (15 days post final restoration) expressed in millimeters and difference of the means from baseline to the end of the treatment (δz). Legend: μ0 = average Crown length at t0; μ1 = average crown length at t1; ε = standard error; δz = Difference between μ0 and μ1.

## Data Availability

Not applicable.
